# Surprisingly Simple Mechanical Behavior of a Complex Embryonic Tissue

**DOI:** 10.1371/journal.pone.0015359

**Published:** 2010-12-28

**Authors:** Michelangelo von Dassow, James A. Strother, Lance A. Davidson

**Affiliations:** 1 Department of Bioengineering, University of Pittsburgh, Pittsburgh, Pennsylvania, United States of America; 2 Howard Hughes Medical Institute Janelia Farm, Ashburn, Virginia, United States of America; Dalhousie University, Canada

## Abstract

**Background:**

Previous studies suggest that mechanical feedback could coordinate morphogenetic events in embryos. Furthermore, embryonic tissues have complex structure and composition and undergo large deformations during morphogenesis. Hence we expect highly non-linear and loading-rate dependent tissue mechanical properties in embryos.

**Methodology/Principal Findings:**

We used micro-aspiration to test whether a simple linear viscoelastic model was sufficient to describe the mechanical behavior of gastrula stage *Xenopus laevis* embryonic tissue *in vivo*. We tested whether these embryonic tissues change their mechanical properties in response to mechanical stimuli but found no evidence of changes in the viscoelastic properties of the tissue in response to stress or stress application rate. We used this model to test hypotheses about the pattern of force generation during electrically induced tissue contractions. The dependence of contractions on suction pressure was most consistent with apical tension, and was inconsistent with isotropic contraction. Finally, stiffer clutches generated stronger contractions, suggesting that force generation and stiffness may be coupled in the embryo.

**Conclusions/Significance:**

The mechanical behavior of a complex, active embryonic tissue can be surprisingly well described by a simple linear viscoelastic model with power law creep compliance, even at high deformations. We found no evidence of mechanical feedback in this system. Together these results show that very simple mechanical models can be useful in describing embryo mechanics.

## Introduction

At the most basic level, morphogenesis depends on mechanics because the mechanical behavior of the embryonic cells and tissues controls how they deform [1]. Therefore, both the physics and biochemical signaling pathways of the embryo contribute to the form of the organism. Recognition that mechanical cues such as substrate stiffness or applied forces can guide cell movement [2], [3], [4], force generation [2], [5], [6], and gene expression [7], [8] has increased interest in the role of physics in morphogenesis.

Several studies suggest that mechanical feedback may play a role in guiding cell behavior and coordinating morphogenesis in the embryo [3], [9], [10], [11], [12], [13], [14], [15]. For example, Odell et al [16] proposed a model in which tension-induced contractility coordinates the timing of apical constriction during ventral furrow formation in *Drosophila*. Consistent with this model, ventral furrow invagination is blocked by mutations that prevent an initial stochastic phase of apical contraction of mesodermal cells [9], and these defects can be rescued by mechanical indentation, indicating that mechanical stimuli can coordinate morphogenetic movements locally. Long-range transmission of forces around the embryo may also be important in coordinating morphogenesis in *Drosophila* [17], [18], [19]: altering the transmission of forces produced by morphogenetic movements in the posterior end of the embryo causes alterations in morphogenetic movements at the anterior end of the embryo [18], as well as changes in expression of the developmental regulatory gene *Twist* [18], [19].

**Figure 1:**
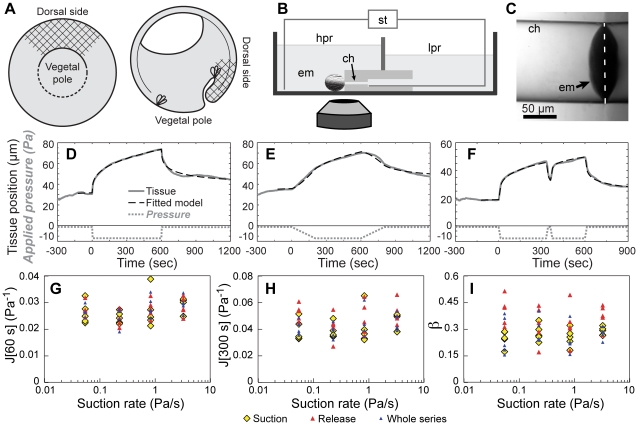
Mechanical response to micro-aspiration was independent of loading rate. A) Diagram of *X. laevis* gastrula (stage 11): vegetal view (left); cross section (right). Hatched areas indicate where measurements were made. B) Diagram of the micro-aspirator (not to scale) on the stage of an inverted microscope. An embryo (em) is pressed to the channel (ch) using a polished glass rod (not shown). The pressures in the high- and low- pressure reservoirs (hpr and lpr) are adjusted hydrostatically. The aspirated tissue is imaged from below. C) Aspirated tissue (arrow) is visible in the channel. The bulk of the embryo is on the right of the channel opening (dashed line) but is hidden by reflections off the channel block surface. D–F) Tissue positions and curve fits using the power-law model for three different pressure histories. G-I) Viscoelastic parameters at different suction rates: compliance at 60 s (G), compliance at 300 s (H) and power-law exponent (I). Different symbols indicate which part of the data were fitted: "suction": −120 to +600 s; "release": +540 to +1200 s; "whole series": −120 to +1200 s.

These studies highlight the need to understand the mechanical behavior of embryonic tissues. To determine whether the complex internal structure and regulation of an embryonic tissue produces complex mechanical behavior of the tissue, we focus on an embryonic tissue in a vertebrate model system, *Xenopus laevis*; specifically, the tissue above the blastopore on the dorsal side of the gastrulating embryo (the dorsal marginal zone; Fig. 1A). This tissue consists of an epithelium covering deeper cell layers [20]. Despite some statistical and technical limitations, one recent study suggests that mechanically stimulated calcium signaling may be important in coordinating cell behaviors in the deep mesodermal layers of this tissue [10]. Here we test whether mechanical stimulation changes the mechanical behavior of the tissue.

Another fundamental challenge is to decipher the relationships between processes that generate force and processes that contribute to viscoelastic resistance in the embryo. Separating these processes is difficult because the same upstream signaling pathways and downstream effectors control both force generation and viscoelastic resistance [21], [22], [23], [24]. Coupling between force generation and cell stiffness is observed in isolated cells [25]. To address this problem we investigate the mechanics of electrically stimulated contractions in the dorsal marginal zone [26]. This approach provides an experimentally tractable proxy for developmentally regulated force generation that is separable from normal developmental controls.

Our goals in this paper are to test the hypothesis that applied forces change the mechanical behavior of a vertebrate embryonic tissue, and to develop tractable, experimentally verified models of the active and passive mechanical behavior of this tissue. To address these issues we first use micro-aspiration to test whether a small-deformation model that treats the embryonic tissue as a homogenous, linearly viscoelastic material is adequate to describe the mechanical properties of the dorsal marginal zone as it undergoes large deformations. In the micro-aspiration method, mechanical properties of a material are calculated based on the deformation of the material as suction is applied to it through a narrow channel [27]. A linear, small deformation model [28] is commonly used in micropipette-aspiration studies despite the fact that such studies typically involve large deformations [28], [29], [30]. Large deformation models of micro-aspiration have been developed for geometries with a large channel diameter to tissue diameter ratio [31], [32], [33], [34], however they do not allow convenient analysis of complex pressure histories. We use a large-deformation finite element model (FEM) to explore the patterns of strain produced by micro-aspiration. We then test two simple mechanical models of induced contractions to identify approaches for measuring force generation. Finally, we test whether tissue stiffness correlates with force generation capacity.

Remarkably, we found that despite the complexity of this embryonic tissue, a small-deformation, linearly-viscoelastic, continuum model appears adequate to describe this tissue's behavior over a 4-fold change in applied forces at large deformations. We found no evidence of mechanical feedback: neither mechanical load nor loading rate detectably altered the mechanical properties of this embryonic tissue. However, this tissue is capable of substantial force generation over short time periods, and its capacity for force generation may be related to its stiffness.

## Results

### Stress application rate does not affect mechanical properties

The viscoelastic properties of cytoplasm may depend on the rate at which it is deformed [35], [36], [37], [38]. Furthermore, cytoplasm can rapidly and dramatically fluidize in response to a suddenly applied, transient load [39]. Therefore we tested whether the rate at which force is applied affects the mechanical properties of an embryonic tissue. We used micro-aspiration (Fig. 1B–C) to test this: suction was applied to a 125 µm diameter patch of tissue located between the blastopore and the equator of the embryo on the dorsal side of intact stage 11 *X. laevis* embryos (∼1400 µm diameter). We calculated the mechanical properties of the tissue from observed displacements. We applied suction (−10.8 Pa) at one of four different rates covering 2-orders of magnitude (from 0.054 to 3.23 Pa/s), and then released the suction at the same rate after 10 minutes (Fig. 1D–E). The time scale of these tests corresponds well to both the time scales of cellular behaviors such as protrusion and contraction that drive morphogenesis at longer time scales in *Xenopus* [40], [41], [42], as well as the time scale of cell divisions which deform surrounding non-dividing cells [43].

We investigated several different linear viscoelastic constitutive equations to identify one that would adequately fit the tissues response at different stress-application rates. Following Sato et al 1990 [28] and Merryman et al 2009 [30] we generalized the linearly elastic half-space model of micro-aspiration developed by Theret et al [44] to use arbitrary linear viscoelastic constitutive equations (compliance depends on time, but not stress) and arbitrary pressure histories using the elastic-viscoelastic correspondence principle (Supplemental Text S1, part 1) [45]. This gives the following, where J(t) is any formulation of creep compliance J(t): 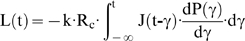
(1)


Here, L is the aspirated length of tissue, k is a constant (k = 0.97; [46]), R_c_ is the channel radius (62.5 µm), t is time, J is the creep compliance function, and P is the applied pressure.

The fit between a simple power-law model of creep compliance and the displacement-versus-time data was quite good, even for complex pressure histories (Fig. 1D–F). Power-law viscoelasticity has been commonly observed in experiments on the mechanical properties of cells [47], [48], [49], [50], [51], [52]. The power law model describes creep compliance, J, as the following function of time, t:
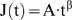
(2)


A and β are fitted parameters. The root mean squared error (RMSE) for the fitted curves were typically small. For the fits to the whole time series (application and release of suction; -120 to +1200 s) the median RMSE was 1.07 µm (min: 0.56 µm; max 2.33 µm); for the fits to the "suction section" (−120 to +600 s) and "release section" (+540 to +1200 s) of the time series analyzed separately, the median RMSEs were 0.47 µm (0.16 to 1.09 µm) and 0.43 µm (0.20 to 1.33 µm). Several other constitutive equations for compliance were tested but the power law model was as good or better than all of them (Supplemental Text S1, part 2).

Although the fit to the linear, small deformation model with power law viscoelasticity was typically quite good, we noticed that during the slowest ramps of pressure (0.054 Pa/s; n = 5) the model predicted a slightly concave-upwards bend during the ramp, whereas the actual displacement versus time during the ramp was always nearly linear (Fig. 1D). This suggests a slight non-linearity of the stress-displacement curve, with higher apparent stiffness at higher strains. This small deviation from the predicted curve was not noticeable in faster ramps of pressure because there were fewer data points during the ramp. An additional caveat is that correlations between fitted parameters for different sub-sections of the same time series were poor. In particular, power-law parameters calculated separately from the "suction section" were uncorrelated with parameters calculated from the "release section". In one clear case, this deviation was driven by slippage of the embryo past the channel opening after suction was released. Slipping, which can occur before application of suction or after release of suction, may have contributed to the poor correspondence in other embryos as well. While there was little clear or consistent pattern to the deviation between fits to the "suction section" and the "release section" there was a trend towards higher values of β and higher calculated compliances at long time scales (J(300 s)) (Fig. 1G–I). Both slippage and deviations from power-law behavior at long time scales would primarily influence fits for the "release section". Thus, all subsequent statistical analyses were done with parameters calculated from the "suction section".

Despite the caveats discussed above, the good fits for different pressure histories, including both ramps of suction (Fig. 1D–E) and pressure pulses (Fig. 1F) allowed us to test whether loading rate affects tissue mechanical properties. We found that the rate of application of suction did not detectably affect calculated mechanical properties (Fig. 1G–I, Table 1), including the power-law exponent (β) and the creep compliances at specific times (J(60 s) and J(300 s)). Since our tissue is insensitive to loading rate over a 60-fold range of loading rates all subsequent experiments used a loading rate of 0.83 Pa/s.

**Table 1:** ANOVA table. Stress application rate vs. viscoelastic parameters.

Factor	J(60)	J(300)	β
Stress rate	*P* = 0.4 (*F*_3,12_ = 1.04)	*P* = 0.4 (*F*_3,12_ = 0.99)	*P* = 0.2 (*F*_3,12_ = 1.67)
Clutch	*P* = 0.07 (*F*_4,12_ = 2.88)	***P*** = 0.04* ( ***F*** **_4,12_ = 3.68)**	***P*** = 0.05* ( ***F*** **_4,12_ = 3.25)**

Analysis of parameters fitted to displacements between −120 s to +600 s relative to initial application of suction. "Clutch", the batch of eggs (collected at the same time from the same mother) from which an embryo was taken, was treated as a random factor, and stress application rate was treated as a fixed factor.

### Embryonic tissue exhibited nearly linear mechanical behavior

To test whether the mechanical properties of the tissue depend on applied stress, we tested whether the measured stiffness changes over a 4-fold range in load pressure. A constant load pressure ranging from −3.6 and −14.4 Pa was applied continuously starting at t  = 0 s. Viscoelastic parameters were calculated from fits to the displacement between −30 and +300 s after application of the load suction. The total aspirated length at 300 s (L(300)) provides an measure of the range of tissue deformations involved. L(300)) is the sum of displacements driven by the load suction, by pressing the embryo to the channel opening, and by the baseline suction. L(300 s) varied by a factor of 2.8 in this experiment: from 24 µm to 66 µm (0.4 to 1.1 times the channel radius).

**Figure 2:**
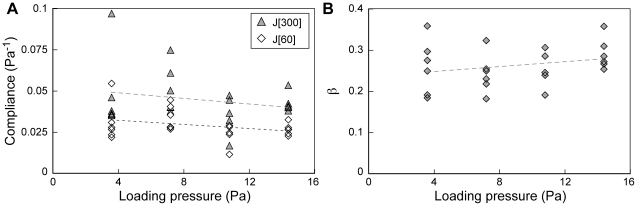
Mechanical response to micro-aspiration is independent of loading pressure. Effect of loading pressure on (A) compliance at 60 s (J[60]) and 300 s (J[300]), and (B) the power-law exponent, β. Lines for least squares fits are shown for visual clarity only. Viscoelastic parameters were calculated from tissue positions between −30 and +300 s after application of loading pressure.

Although there was a slight trend of increasing tissue stiffness with increasing suction, it was not statistically significant (Fig. 2A; Table 2). Furthermore, the exponent of the power-law model did not change significantly over the range of pressures used (Fig. 2B). Hence, the mechanical properties of the material appear remarkably linear over a broad range of stresses and strains.

**Table 2:** ANCOVA table. Loading pressure vs. viscoelastic parameters.

Factor	J(60)	J(300)	β
Clutch	*P* = 0.07 (*F*_5,11_ = 2.85)	***P*** = 0.01* ( ***F*** **_5,11_ = 4.90)**	*P* = 0.2 (*F*_5,11_ = 1.79)
Pressure	*P* = 0.1 (*F*_1,11_ = 3.20)	*P* = 0.15 (*F*_1,11_ = 2.42)	*P* = 0.2 (*F*_1,11_ = 1.80)
Pressure*Clutch	*P* = 0.4 (*F*_5,11_ = 1.18)	*P* = 0.15 (*F*_5,11_ = 2.05)	*P* = 0.7 (*F*_5,11_ = 0.65)

Analysis of parameters fitted to displacements between −30 s to +300 s relative to application of loading pressure. Clutch and Pressure*Clutch were treated as random factors, while loading pressure was treated as a linear covariate.

### Strains experienced during micro-aspiration

**Figure 3:**
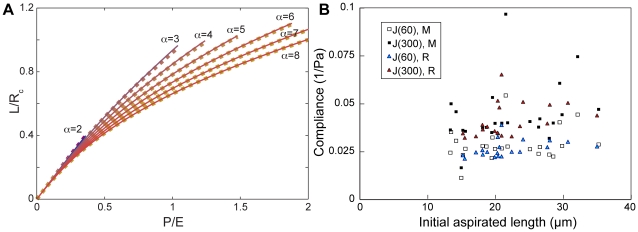
The effect of non-linear material properties. (A) Aspirated length L as a function of pressure P in an FEM model for different degrees of material non-linearity (increasing α). Aspirated length was normalized to channel radius, and pressure was normalized to the Young's modulus (‘E’). Solid lines: frictional coefficient of 0; dotted lines: frictional coefficient of 0.5. (B) There was no detectable effect of initial aspirated length on the measured compliance for either the loading rate experiment (‘R’, triangles) or the load magnitude experiment (‘M’, squares).

The biological relevance of our experiments depends on the correspondence between the magnitude and rate of the experimentally applied deformations and the deformations during morphogenesis. Therefore, we used an explicit large-deformation elastic FEM model with a one term Ogden material model [53] to investigate how the patterns of deformation vary with aspirated length. Viscoelastic effects should not substantially alter the stretch patterns at a given deformation. We used a Poisson ratio of 0.4, within the measured range for cells [54], [55]. The tissue often slips past the channel opening prior to application of the loading suction, indicating that friction between the embryo and the channel block is low. We considered the effect of friction by varying the frictional coefficient from 0 to 0.5. In the FEM model, the effect of friction on the aspirated length versus pressure curve was negligible (Fig. 3A). As expected, increasing the degree of strain hardening (increasing α) made the pressure-deformation curve increasingly non-linear, however the deviation from linearity was only prominent for large differences in aspirated length (Fig. 3A).

We were concerned that variation in initial aspirated length (L(0)) might mask effects of non-linear material properties. The initial aspirated length is produced by the baseline suction and the light compression necessary to form a seal on the embryo. If tissue non-linearity was high, then higher initial aspirated lengths would be associated with lower compliances. However we found no such effect (Fig. 3B; Supplemental Text S1, part 3), indicating that the variation in initial aspirated length did not detectably mask non-linearity in the pressure-deformation curve.

The slope of the aspirated length versus pressure line for a neo-Hookean material (α = 2; L/R_c_ = 1.107P/E; Fig. 3A) is higher than predicted for an infinite thickness sample (k = 0.97), but is close to the predicted slope (1.13) expected for the modeled sample tissue thickness based on previous small deformation models of finite thickness materials [46], [56]. The difference in slope between our model and the infinite thickness model (used in our experimental analyses) is negligible for our purposes. Our model uses a corner radius of the channel opening (the "fillet radius") of 0.02R_c_, an upper limit on the expected corner radius of our channels based on optical microscopy. Previous large deformation models indicate that the corner radius can affect time- and pressure-dependence of the aspirated length [31], [32]. However, the close match between our model predictions for α = 2 and the small deformation model predictions [46], [56] indicates that decreasing the modeled corner radius below 0.02R_c_ should not substantially alter the predicted aspirated length for our geometry.

**Figure 4:**
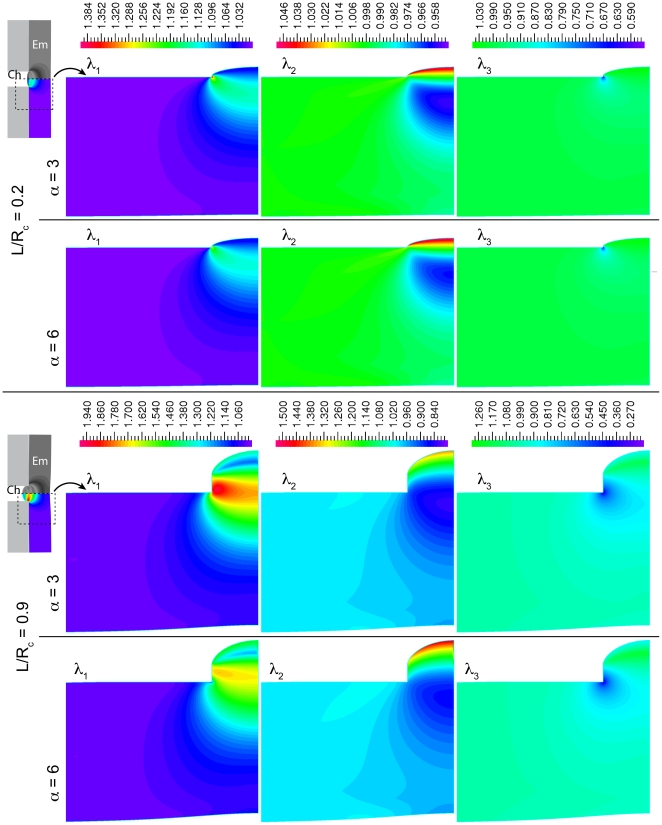
Micro-aspiration produces complex patterns of stretch and compression. Maps of the three principal stretch ratios, λ_i_, for different values of α and different aspirated lengths ‘L’ (relative to channel radius, ‘R_c_’), and no friction between the tissue and the channel. Only half of the channel is shown because the model was axisymmetric. The plots were cropped as indicated by dotted lines in the insets (‘Ch’: channel; ‘Em’: embryo). Deformations outside of the enlarged region were low and nearly uniform. Note that the color scales differ for different principal stretches, and for different aspirated lengths.

The distribution of stretch within the aspirated tissue was qualitatively similar across a wide range of aspirated length (Fig. 4). Stretch ratios are a convenient measure of deformation at large strains. They are defined as the ratio of the deformed length to the undeformed length of a part of the material. The distribution of stretch resembles previous reports using low deformation models [56]. For low aspirated lengths (L/R_c_ = 0.2; α = 3) the first principal stretch ratio was high in a ring near the channel opening edge and much lower elsewhere (Fig. 4). The core of the aspirated tissue exhibited moderate stretches, and both the material near the surface of the aspirated tissue, and the large volume of deeper tissue was stretched or compressed by much smaller amounts. At larger aspirated lengths (L/R_c_ = 0.9) the model exhibited much the same pattern except with larger peak stretch ratios and a greater fraction of the tissue under high stretch or compression (Fig. 4). For α = 6, the results were qualitatively similar but the stretch ratios were closer to 1. Due to the Poisson effect, the high stretch region was also associated with substantial compression along the third principle axis, however some of the material was stretched along all three axes (Fig. 4). Reducing the corner radius should increase the predicted stretch and compression in the material near the opening edge, however it should not substantially alter the stretches far from the opening edge or change the qualitative pattern of deformation. Our results indicate that aspirated tissue in our experiments experiences a wide range of strains at any given aspirated length.

### Reconstructing pressure histories

**Figure 5:**
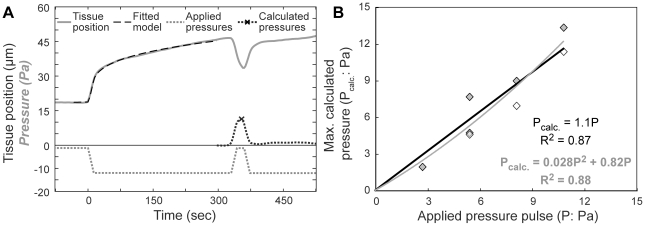
Pressure time courses can be reconstructed from displacements. A) An example of an applied pressure pulse (see Fig. 1F). The viscoelastic model was fitted to the tissue position vs. time data prior to the pressure pulse (dashed line) given the applied pressure (dotted gray line). The fitted viscoelastic parameters were then used to calculate subsequent pressure changes (black dotted line) from tissue displacements allowing comparison of applied and calculated pressure pulse. B) The magnitude of actual applied pressure pulses versus the maximum pressure during the pulse calculated based on the viscoelastic model.

Since one goal in adopting a linear viscoelastic model is to understand the coupling between stiffness and contraction forces we wanted to know whether our model and analytic framework could accurately reconstruct forces during an experimentally controlled perturbation. Therefore, we asked whether we could use this model to correctly extract the magnitude of applied pulses of pressure (Fig. 1F, 5A). We applied a fixed load pressure (−10.8 Pa) from 0 to 330 s, and then raised the pressure by 25% to 100% of the load pressure at +330s, finally returning to the original load pressure at +360 s. We used the initial response to the load pressure (−30 to +300 s) to calculate parameters of the power-law viscoelastic model for each embryo. We then used these parameters to extrapolate what the tissue position would have been at later time points if there had been no further pressure changes. Finally, we used the differences between the actual tissue positions and the extrapolated tissue position to calculate pressure changes occurring after +300 s (Supplemental Text S1, part 4; Fig. 5A).

The magnitude of the calculated pressure pulse was tightly correlated to the applied pressure pulse, with a slope close to unity (Fig. 5B). Adding a quadratic term did not improve the fit (Fig. 5B). This shows that the linear, small-deformation model can reconstruct forces driving tissue movements despite the large deformations that occur during micro-aspiration. Therefore, we use this approach to estimate the forces driving induced contractions (below).

### Mechanics of induced contractions

Much as artificial stimulation of muscle allowed tightly controlled tests of muscle behavior, we expect that the capacity to acutely induce contraction of embryonic tissue will be useful in separating active from passive mechanical behaviors of these tissues. Here we use electrically stimulated contractions to test whether two mechanical models of the behaviors driving contractions can predict the behavior of the tissue as a function of applied suction. We used a 4 ms, 2.6 µA current pulse as a stimulus to induce contractions in the aspirated tissue (Supplemental Text S1, part 5).

Our first model proposes that contractions are driven by the development of tension in a layer of material near the apical surface ("apical contraction model"; Supplemental Text S1, part 6.1). Because we used the same stimulus for all load pressures, this model predicts that the apical tension will be independent of load pressure. We assume this apical tension is not present before the stimulus. A thin membrane under tension would exhibit softening with increasing load suction or increased initial aspirated length [27]. Therefore this assumption is justified by the observation that stiffness is independent of load suction and initial aspirated length with, perhaps, slight stiffening at higher loads.

The induced apical tension (T) is equivalent to a surface tension that generates a time varying pressure term ("equivalent pressure", P_eq._) in addition to, and counter to, the loading pressure. The equivalent pressure drives the contraction against the viscoelastic resistance of the embryo. To estimate the equivalent pressure we calculated the time course of pressures one would need to apply to mimic the changes in aspirated length during a contraction using the method described above for reconstructing applied pressure pulses (Supplemental Text S1, part 4). We then calculated the apical tension based on Laplace's law, using the aspirated length and the radius of the channel to estimate the radius of curvature (r) of the aspirated tissue based on the assumption that the tissue surface can be approximated as a spherical cap (Supplemental Text S1, part 6.1): 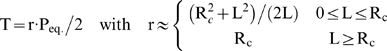
(3)


This estimate of the radius of curvature incorporates errors due to lumpiness and asymmetry in the tissue [43] and the non-spherical shape expected based on the FEM model (Fig. 4). However, the estimate appears to be a reasonable first approximation (Supplemental Text S1, part 6.1). This model predicts that the equivalent pressure would increase with increasing load pressure because the radius of curvature of the tissue would decrease as the aspirated length increases.

In our second model we propose that contractions are driven by a uniform cell-generated isotropic stress developed everywhere in the tissue ("isotropic contraction model"; Supplemental Text S1, part 6.2). This model requires the tissue to be compressible (Poisson's ratio, ν<0.5). Previous studies suggest that cells have a Poisson ratio between 0.3 and 0.5 [54], [55]. A simple model suggests that the ratio of the maximal displacement during the contraction (m) to the aspirated length before the contraction (L[300]) would be independent of the applied pressure (Supplemental Text S1, part 6.2). Because this model predicts that the displacement during contraction increases with increased aspirated length, this model also predicts that the equivalent pressure for the contraction will increase with increased load pressure.

**Figure 6:**
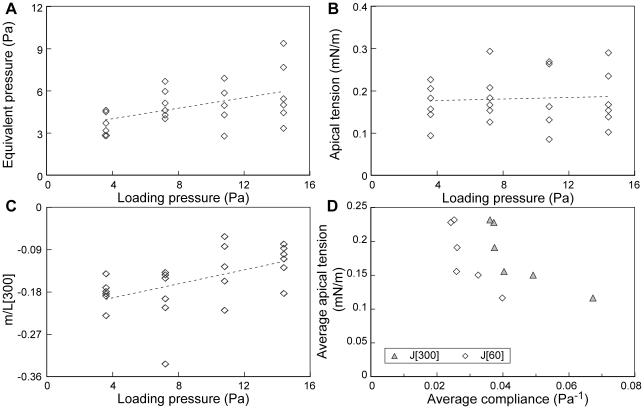
Comparing two models of induced contractions. Loading pressure versus magnitude of induced contractions calculated as A) equivalent pressure, B) apical tension, or C) the ratio of the maximal displacement during the contraction, ‘m’, to the pre-contraction aspirated length, L[300]. Lines for linear least squares fits are shown for clarity only. D) Average compliance for each clutch versus average apical tension for each clutch. Compliance was calculated at 60 s (J[60]) and 300 s (J[300]); n = 3 to 4 for each clutch.

Of these two models, our results are consistent with the apical contraction model, and are inconsistent with the isotropic contraction model. As predicted by both models, the maximal equivalent pressure during contractions increased significantly with increasing suction (Fig. 6A, Table 3). Log-transformation reduced the apparent differences among treatments in the variance of equivalent pressure, but the dependence on loading pressure remained statistically significant (Table 3). As predicted by the apical contraction model, the maximal surface tension was independent of suction (Fig. 6B, Table 3). However, contrary to the prediction of the isotropic contraction model, the ratio of contraction displacement (m) to pre-contraction aspirated length (L[300]) varied significantly with loading pressure (Fig. 6C). Given the success of the apical contraction model, apical tension appears to be a better measure of contraction strength than equivalent pressure.

**Table 3:** ANCOVA table.

Factor	Equivalent pressure	Ln[Eq. pressure]	Apical tension	m/L[300]
Clutch	*P* = 0.2 (*F*_5,11_ = 1.66)	*P* = 0.2 (*F*_5,11_ = 1.61)	*P* = 0.2 (*F*_5,11_ = 1.88)	*P* = 0.3 (*F*_5,11_ = 1.32)
Pressure	***P*** = 0.008* ( ***F*** **_1,11_ = 10.5)**	***P*** = 0.01* ( ***F*** **_1,11_ = 9.69)**	*P* = 0.7 (*F*_1,11_ = 0.11)	***P*** = 0.01* ( ***F*** **_1,11_ = 9.06)**
Pressure*Clutch	*P* = 0.075 (*F*_5,11_ = 2.75)	*P* = 0.1 (*F*_5,11_ = 2.19)	*P* = 0.3 (*F*_5,11_ = 1.52)	*P* = 0.4 (*F*_5,11_ = 1.07)

Loading pressure vs. contraction strength. Clutch and Pressure*Clutch were treated as random factors, while loading pressure was treated as a linear covariate.

### Contractility and tissue stiffness

Using the models developed above, we tested for a relationship between tissue stiffness and the capacity of the same tissue to generate force during electrically induced contractions. We first analyzed each contraction as a separate datum with stiffness (measured as the compliance at 300 s, J(300)), as a covariate, and clutch, and clutch-stiffness interactions as separate random factors in an ANCOVA. We found no effect of stiffness on apical tension when considering embryos individually (*P* = 0.7, *F*_1,11_ = 0.17). However, we noted that the compliance showed significant variation among batches of embryos (clutch-to-clutch variation; Tables 1 & 2). This suggested that using clutch as a factor in the analysis may have hidden any correlation between stiffness and contractility. Therefore we tested for a correlation between average stiffness and average force generation among clutches. We found that stiffer clutches (lower average compliance) produced stronger contractions (higher average maximal surface tension; Fig. 6D; J(60): *P* = 0.04, τ_b_ = −0.733; J(300): *P*≤0.01, τ_b_ = −1; Kendall's Tau test for correlations).

## Discussion

### Simple mechanics from a complex tissue

Most studies on mechanical feedback in embryos have focused on tissues that generate forces to drive morphogenesis. This raises a question of whether mechanical stimuli alter the mechanical behavior of embryonic tissues that are not actively deforming their surroundings. If it does, it would suggest that the whole embryo is involved in a complex system of mechanical feedbacks and it would raise the question of how these long-range feedback processes are coordinated to produce localized shape changes [11], [13].

Here we used the micro-aspiration approach to investigate the mechanical properties of the dorsal marginal zone of the gastrulating frog embryo. Even though the dorsal embryonic tissue is extremely complex, with obvious heterogeneity, and it is capable of active forces generation either stochastically [43] or following exogenous stimulation [26], its aggregate mechanical behavior was surprisingly simple. Despite the large deformations involved in our tests, the behavior of this tissue was remarkably well described by a linear, small-deformation model. We found no evidence that the mechanical properties of this tissue are affected by either the magnitude of stress or the rate of stress application over the broad range of stresses and stress-application rates tested. Note that we can detect relatively small changes in stiffness using this approach (20%), despite the high degree of embryo-to-embryo variation in tissue mechanical properties [43]. Furthermore, the time scales considered here bracket the time scales suggested for mechanical feedback by some studies in *Xenopus* [10] and *Drosophila* [14], [15]. Consistent with our previous results [26], [43] we found no evidence that mechanical stimulation induced contractions.

It is perhaps surprising that this tissue shows little or no sign of mechanically induced contractility [26], [43] or mechanically induced changes in stiffness (this study) given that a recent study argues that mechanical stimuli induce short (<1 min) calcium pulses in *Xenopus* mesodermal cells [10] and calcium waves are associated with contractions [57]. We would expect these cells would be strongly stimulated by micro-aspiration given the predicted patterns of strain. In one experiment, Shindo et al [10] touched a group of cells with a polished glass rod and noted calcium transients, however as best as we can determine, data from only one explant was shown and wounding of the cells by adhesion to the glass was not ruled out. Interestingly, the time scale of the calcium transients (6 to 45 s) was similar to the time scale (10 to 90 s) of contractions observed following stimulation of the epithelium with a laser or electrical pulse, or with cell lysate [26]. Shindo et al [10] also interpreted an increase in calcium transients in cells that crawled under grooves in a barrier as evidence for mechanical stress-induced calcium release, however the mechanical stimulus associated with that experiment was uncharacterized. The discrepancy between Shindo et al's [10] study and our own highlights the fact that biochemical changes need not reflect mechanical changes, and highlights the necessity of ruling out non-mechanical wounding effects in studies of mechanical signaling [26], [58].

While previous studies have not systematically characterized the viscoelastic properties of an embryonic tissue over a wide range of loads and loading rates, our results are consistent with previous studies on other embryonic tissues using tensile, compressive, and indentation tests [59], [60], [61], [62]. The slight increase in stiffness with increasing stress that we observed is small relative to what we would expect from the strain-stiffening behavior of cells [63] and cross-linked actin gels [64]. While the observed stiffening was not statistically significant, it is corroborated by the small but consistent deviations between the fitted curves and the displacement data at low stress application rates. Note that this slight stiffening with increasing suction suggests that the epithelium does not behave like a liquid-like drop with a surface tension or like a thin shell under internal pressure, which would appear to soften with increasing suction [27].

How do the strains we applied compare to endogenous strains? Although morphogenetic movements are slow, the individual cell behaviors such as protrusions and contractions that drive them can be quite fast and localized [40], [42]. For example, the apical surfaces of bottle cells contract by up to 20% in area over 5 minutes [42], requiring concomitant stretches elsewhere to maintain cell volume. Dividing cells also dramatically and rapidly stretch their neighbors in the epithelium [43] by up to 10 to 30% in 4 min. Based on our FEM model, the aspirated tissue would experience a wide range of deformations at any given aspirated length (Fig. 4). Although some parts of the tissue would experience very high deformations even at low aspirated lengths, the bulk of the tissue would experience considerably less deformation, comparable to the endogenous stretches associated with the cellular behaviors noted above. Given the time scales of our tests and the viscoelasticity of the tissue, it would reach these stretches by ∼5 min, comparable to the endogenous timescales. While both the complex distribution of strains in the aspirated tissue and the time scale of our tests do limit our ability to rule out some possible models of strain-sensitivity, our results put sharp limits on the form, the time scale, and the spatial scale of any such hypothetical response.

Our results here, together with previous experimental [21], [43], [59], [60], [61], [62] and theoretical [65] studies, suggest that relatively simple constitutive laws may suffice to describe the bulk behavior of embryonic tissues. Although the embryonic tissue is certainly complex and non-linear, simple models with only a few measurable parameters may be more useful than complex models given the high degree of variability of embryo mechanical properties [43], [59], [60], [66], [67], [68], [69]. Much of this variability appears to reflect real embryo-to-embryo or clutch-to-clutch variation rather than experimental noise [43], [67], [69]. Given that human engineers find it easier to control linear systems than non-linear systems, we suspect that the simplicity of this tissue's mechanical behavior simplifies the control of the morphogenesis for the embryo as well.

### Induced contractions

Our results indicate that micro-aspiration can be used to test simple mechanical models of contraction. Specifically, our results are consistent with an apical contraction model, but not an isotropic contraction model, of electrically induced contraction. A limitation is that our models assume that tissue viscoelasticity remains constant during a contraction. At present we cannot test this assumption, but we know that changes in F-actin distribution occur in parallel with electrically induced contraction [26], and that F-actin affects embryo stiffness [59]. In future work we hope to implement feedback control of the pressure that will allow us to relax this assumption.

We previously hypothesized that stiffer tissues might generate higher forces in order to explain the robustness of gastrulation to substantial natural variation in tissue stiffness [43]. This is consistent with observations that myosin activity contributes strongly to both force generation and stiffness in embryos [23], [24], [59]. However, in an earlier study we found no relationship between natural variation in stiffness and natural variation in force generation during contractions [26]. Our results here – using a larger data set and a model of contractions that takes into account viscoelasticity – suggest that force generation increased with increasing stiffness among clutches: softer clutches appeared to produce lower apical tensions during contractions.

### Summary

The *Xenopus* gastrula dorsal embryonic epithelium (and underlying cells) is describable by a simple linear viscoelastic model over a large range of stress and strain. We found no evidence of mechanically induced changes in the mechanical properties of the tissue. This simple model, in concert with electrical stimulation, allows estimation of the magnitude of forces produced during exogenously induced contractions.

## Methods

### Embryo handling

Animals used in this study were treated according to an animal use protocol issued to Dr. Davidson (IACUC Protocol #: 0903349) that has been reviewed and approved by the University of Pittsburgh Institutional Animal Care and Use Committee (Assurance #: A3187-01) in order to meet all US government requirements.

Eggs were collected, fertilized, and de-jellied following standard methods [70]. Embryos were staged following the Nieuwkoop and Faber staging tables [71]. Embryos were cultured in 0.33 x Modified Barth's Solution (MBS) until stage 9 (late blastula) when the vitelline membranes were removed with great care to minimize wounding. Measurements were done at stage 11 (mid-gastrulation). During and after vitelline removal, embryos were maintained in 0.33 x MBS with 2 mg/mL bovine serum albumin ("BSA"; Sigma Aldrich), and 8 µL/mL of antibiotic-antimycotic (A5955; Sigma-Aldrich, St. Louis, MO). BSA was added to reduce adhesion of the embryo to the measurement apparatus. Measurements were made at 20 to 22°C.

### Microaspiration

Our apparatus and micro-aspiration methods have been described previously [26], [43]. Briefly, embryos are gently pressed onto the opening of a 125 µm diameter channel through a polydimethylsiloxane (PDMS) block. The pressure difference across the channel was controlled hydrostatically using a computer-controlled piston to change the water level. Drift in the system was measured based on the change in piston position needed to stop the movement of particles in the channel at the end of the measurement. The drift appeared to be due primarily to evaporation and was typically within ± 3% of the loading suction, although it is occasionally more substantial. In a typical test, a baseline pressure of −1.2 Pa was applied about 5 to 7 minutes before the loading pressure was applied (defined as t = 0). Applying a baseline pressure was done to test the seal and improve the clarity of the image. Imaging was described previously [26]. Tracking of the tissue boundary was done with a custom macro in ImageJ [72]. The macro uses a Canney-Deriche filter and hysteresis thresholding (http://imagejdocu.tudor.lu/) to identify the tissue edge. For experiments involving calculations of "equivalent pressure" a 3-point moving average filter, implemented in Matlab 7.8, was used to reduce the noise in the tissue displacements due to pixelation.

In the experiments testing the effect of loading rate or load pressure, we tested a single embryo from each of 5 to 6 clutches at each treatment (specified loading rate or specified load pressure). Data from one embryo in the load pressure experiment was not analyzed because a contraction began just prior to the application of the load pressure. In the pressure pulse experiment 3 to 5 embryos were tested for each of 2 clutches. The order of treatments was randomized for each clutch in all experiments.

### Electrical stimulation

The basic protocol for electrical stimulation was modified from Joshi et al 2010 [26]. Current was provided by Platinum-Iridium electrode (A–M Systems, Inc. Carlsborg, WA) placed in the micro-aspirator channel (3 to 4 mm from the embryo) and a Platinum-Iridium counter electrode in the bath with the embryo. Electrode position made no detectable difference to the measured current. Electrical current was provided with a stimulator (WPI A320; World Precision Instruments, Sarasota FL). Since the resistance of the media was high and only low currents were needed to stimulate the tissue, we could not use this stimulator directly as a constant current source. Therefore we ran the stimulator across a 10 kΩ resistor in parallel with the channel and placed a 505 kΩ resistor in series with the channel. This allowed greater control over the current through the channel. We measured the current using an Oscilloscope (DPO3014; Tektronix, Beaverton, OR) attached across the 505 kΩ resistor. For most tests we placed an additional 6.4MΩ resistor in series with the channel to minimize variation in the current. The 6.4 MΩ resistor was removed when we tested for variation in the added resistance of the embryo (Supplemental Text S1, part 5). This simple set up allowed us to give consistent, nearly square current pulses (2.6 µA; channel negative with respect to embryo) with a duration of 4 ms (Supplemental Text S1, part 5).

### Data analysis

By treating the baseline pressure as a step function and subsequent pressure changes as a series of ramps with slopes w_i_ we obtain the following function using the power law model of compliance, and the generalized linear viscoelastic model for micro-aspiration given in equation 1 (Supplemental Text S1, part 1): 
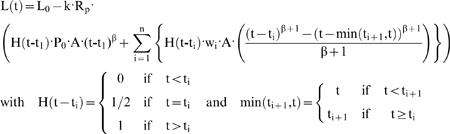
(4)


Here, L_0_ is a fitted parameter to account for initial compression and A and β are fitted parameters of the power-law viscoelastic model.

To fit the displacement data from the time-lapse videos to the viscoelastic model, we used Matlab 7.8's (The MathWorks, Inc) implementation of constrained minimization to minimize the sum of squared residuals between the viscoelastic model and the displacement data. Compliances at 60 s or at 300 s were calculated from the fitted viscoelastic parameters. Statistical tests on the fitted power-law exponent and the calculated compliances were done in SPSS version 16 for Windows.

### Finite element model

The finite element simulations were performed with commercial software (ADINA 8.6.1) using a two-dimensional model assuming axisymmetry. As stress is strongly concentrated near the channel opening, the model geometry far from the channel should have little effect on the aspirated length. Small deformation models indicate that – for tissue thickness beyond 2R_c_ – tissue thickness has little effect on the aspirated length [46], [56]. Therefore, the thickness and radius of the tissue sample were taken as 3R_c_ and 9R_c_, within the range of values for the dorsal marginal zone. We were unable to precisely resolve the channel opening corner radius of the channel opening using light microscopy, but it appeared to be <0.02R_c_, so we used a corner radius of 0.02R_c_ in our simulations. To preserve axisymmetry, the center of the tissue sample was constrained to have zero displacement perpendicular to the channel axis. The surface of the tissue that spans the channel opening was subject to a prescribed normal pressure. All other surfaces of the tissue sample were unconstrained. Contact between the tissue and the channel was modeled using a constraint-function contact model. Two variants of the contact were modeled: in the “friction-less” case the tissue slid along the channel with zero resulting stress, while in the “adherent” case the tissue was subject to a relatively large Coulombic friction (µ = 0.5). The mesh itself was constructed from approximately 40,000 4-node quadrilateral elements (Supplemental Text S1, part 7). The sensitivity of our results to mesh density was examined by recasting the mesh with 20,000 elements, the resulting aspirated displacements were found to be within 0.5% of those in the more refined mesh.

To maintain robust convergence, the model was run as an implicit-dynamics simulation in which the pressure was increased stepwise. After each step increase in pressure, the pressure was held constant for a duration that allowed the model to fully relax to the static configuration (<1% subsequent change). As such, even highly deformed geometries could converge to a solution. To further aid convergence, the tissue material model incorporated viscoelasticity that provided a damping force to the system. This viscoelasticity took the form of a 2-element generalized Maxwell material model extended to large stains with the Holzapfel formulation [53]. The stiffness of the viscoelastic element was 10 times the stiffness of the static element, and the decay period of the viscoelastic element was 2% of the pressure hold time. Using dynamic analysis and modest viscoelastic damping, the models converged consistently with even large displacements. However, it must be emphasized that since the model is always allowed to fully relax back to the static configuration between increases in pressure, the calculated aspiration displacements always reflect those of a static load.

In order to provide insight into the potential range of stress-strain distributions, and the sensitivity of aspirated displacement to material non-linearity, simulations were performed with a series of material models based on ADINA's standard implementation of a single-term Ogden model [53], [73]. The strain energy density, W, is given as follows: 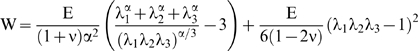
(5)


At the small deformation limit, E is the Young's modulus and ν is the Poisson's Ratio. The λ_i_ are the stretch ratios. The parameter α controls the non-linearity of the material properties. The Young's modulus at infinitesimal strain was held constant while the α was varied between values of 2 and 8. An Ogden model with α = 2 is exactly equivalent to a Neo-Hookean material model, while an Ogden model with α = 8 represents a sharply increasing tangent modulus with strain.

## Supporting Information

Text S1 Supplemental models and data in seven parts.
(PDF)
