# Supplementary material for: Surprisingly Simple Mechanical Behavior of a Complex Embryonic Tissue
Source: PLoS One. 2010 Dec 28;5(12):e15359. doi: 10.1371/journal.pone.0015359 (PMC3011006; doi:10.1371/journal.pone.0015359)
Supplement: Text S1 — Supplemental models and data in seven parts. (PDF) [file pone.0015359.s001.pdf]

SUPPLEMENTAL TEXT: Surprisingly simple mechanical behavior of a complex embryonic tissue

Contents

2-5 (Part 1) Generalized linear viscoelastic model of micro-aspiration

6 (Part 2) Alternative viscoelastic constitutive equations

7 (Part 3) Test for effect of initial aspirated length on apparent compliance

8-9 (Part 4) Reconstructing pressures from displacements

10-12 (Part 5) Electrically induced contractility

13-18 (Part 6) Two models of tissue contractions

19 (Part 7) Finite element mesh

20 Literature cited

(Part 1) Generalized linear viscoelastic model of micro-aspiration

Here we adapt Sato et al's (1990) model to use with complex pressure histories (Merryman et al., 2009) and arbitrary linear viscoelastic material models. Sato et al (Sato et al., 1990) used the elastic-viscoelastic correspondence principle to develop a standard linear solid (SLS) viscoelastic model as an extension of the small-deformation, linearly-elastic continuum model of micropipette-aspiration developed by Theret et al (Theret et al., 1988). The half-space model assumes the tissue can be approximated as an infinitely thick and infinitely wide block. The thickness of the tissue at the investigated location is sufficient to be approximated by the half-space model, although heterogeneity of the tissue likely causes deviations from the model (von Dassow and Davidson, 2009). The linearly-elastic half-space model gives the aspirated length L at time t as follows (Theret et al., 1988; Boudou et al., 2006):

(eqn S1.1)
$$L(t) = -\frac{k \cdot R_c \cdot P(t)}{E}$$

Here E is the Young's modulus, P is pressure and R_c is the channel radius. The parameter k is a function of the thickness of the wall of the channel. For our case with a very thick wall, it will be approximately 0.97.

To convert the linear half-space model for use with arbitrary linear viscoelastic material models we use the elastic-viscoelastic correspondence principle following Sato et al (1990). The correspondence principle is only applicable when the nature of the boundary conditions (i.e. whether they have specified stress or specified displacement) is constant with time (Findley et al., 1989). We suspect this assumption may be violated in micro-aspiration since the aspirated material can move over the lip of the channel

opening. However Sato et al's (Sato et al., 1990) model has been used widely and appears to be a reasonable first approximation.

We replace the constant E by the Laplace transform of the time dependent relaxation modulus $E_L(s)$ multiplied by the transform variable s, and we replace L(t) and P(t) by their Laplace transforms, $L_L(s)$ and $P_L(s)$ (Findley et al., 1989):

(eqn. S1.2) $$L_L(s) = -\frac{k \cdot R_c \cdot P_L(s)}{s \cdot E_L(s)}$$

Since we apply a controlled load to the tissue rather than a controlled strain, we make the following substitutions to use the Laplace transform of creep compliance ($J_L(s)$) instead of the relaxation modulus:

(eqn. S1.3) $$J_L(s) = \frac{1}{s^2 \cdot E_L(s)}$$

This gives the Laplace transformed aspirated length in terms of the Laplace transformed compliance:

(eqn. S1.4) $$L_L(s) = -k \cdot R_c \cdot P_L(s) \cdot s \cdot J_L(s)$$

Assuming the pressure is 0 at time 0, the inverse Laplace transform gives the following equation for any general linear viscoelastic material with creep compliance J(t):

(eqn. S1.5) $$L(t) = -k \cdot R_c \cdot \int_0^t J(t-\gamma) \cdot \frac{dP(\gamma)}{d\gamma} \cdot d\gamma$$

Note that one can shift the time scale so that the lower limit of integration occurs at arbitrarily negative times. This generalizes the model to allow the use of arbitrary pressure histories and arbitrary linear viscoelastic constitutive equations for creep compliance.

Although large deformation viscoelastic models have been developed (Zhou et al., 2005), they are limited to specific viscoelastic formulations and specific pressure

histories. The large deformation models that have been developed so far assume that the channel is large relative to the width of the aspirated tissue. Furthermore, they do not provide convenient formulae to use in the analysis of experimental data.

In our experiments pressure changes occurred as a series of ramps. Application of the loading suction (defined as t=0) began at 5 min after the start of the video (t=-300), and it was released after 10 min (t = 600 s). We approximated the application of the small baseline suction as a step function at t_1= - 300 s, although in practice it was typically applied between 30 s and 90 s before the start of the video (t=-390 to -330 s). Because the pressure drift appeared to be due to evaporation, it was accounted for by subtracting a linear change in pressure from the applied pressure history. The effect of including the drift term was typically negligible. Hence the derivative of pressure changes with time was as follows:

(eqn. S1.6)
$$\frac{dP(t)}{dt} = \begin{cases} P_0 \cdot \delta(t - t_1) & t \le t_1 \\ w_1 = \frac{(P_2 - P_1)}{(t_2 - t_1)} & t_1 < t \le t_2 \\ w_2 = \frac{(P_3 - P_2)}{(t_3 - t_2)} & t_2 < t \le t_3 \\ \vdots & \vdots \\ w_n = \frac{(P_{n+1} - P_n)}{(t_{n+1} - t_n)} & t_n < t \le t_{n+1} \end{cases}$$

Here, the w_i terms are slopes of the pressure change with time, and $\delta(t-t_1)$ is the Dirac Delta function (the derivative of a step function). The slopes were determined from the piston movement and combined with the drift. These derivatives were substituted into eqn. S1.5, along with the appropriate form of creep compliance for each model.

To account for the effect of pressing the embryo on to the channel opening, we added a baseline displacement, L_0, as a fitted parameter to the model. Therefore, the final form of the fitted model was as follows:

(eqn. S1.7)

$$L(t) = L_0 - k \cdot R_c \cdot \left(H(t-t_1) \cdot P_0 \cdot J(t-t_1) + \sum_{i=1}^{n} H(t-t_i) \cdot w_i \cdot \int_{t_i}^{\min(t_{i+1},t)} J(t-\gamma) \cdot d\gamma \right)$$

For power-law viscoelasticity (main text equation 2), this finally gives:

(eqn. S1.8)

$$L(t) = L_0 - k \cdot R_c \cdot \left(H(t-t_1) \cdot P_0 \cdot A \cdot (t-t_1)^{\beta} + \sum_{i=1}^{n} \left\{ H(t-t_i) \cdot w_i \cdot A \cdot \left(\frac{(t-t_i)^{\beta+1} - (t - \min(t_{i+1},t))^{\beta+1}}{\beta+1} \right) \right\} \right)$$

with $H(t-t_i) = \begin{cases} 0 & \text{if } t < t_i \\ 1/2 & \text{if } t = t_i \\ 1 & \text{if } t > t_i \end{cases}$ and $\min(t_{i+1},t) = \begin{cases} t & \text{if } t < t_{i+1} \\ t_{i+1} & \text{if } t \geq t_i \end{cases}$

Here A, and β are fitted parameters from the power law model. Including the initial compression as a fitted constant (L_0) is a reasonable simplification based on observation that embryos that were compressed with no added pressure frequently showed fairly constant aspirated lengths (although contractions and tissue rolling occurred in other embryos).

(Part 2) Alternative viscoelastic constitutive equations

Several alternative viscoelastic constitutive equations were tested by visual inspection, but none worked as well as the power law model for the tested time scale. Two-parameter models consisting of a single viscous element and a single elastic element either in series (a Maxwell model) or in parallel (a Kelvin model), failed to fit the data. We tested 3- and 4- parameter models including a standard linear solid model, a Burger's model, a double exponential model, and a power-law model with an elastic element added in series. None of the more complex models presented an obvious advantage in terms of how well they could fit the data: all of these models (including the simple power law model) could fit the data from each embryo, although some models worked better with some embryos than with others. However, the power-law model, converged on a good fit much more reliably than any more complex model. All starting parameters converged to the same good solutions with the power-law model. However, starting parameters had to be carefully chosen for more complex models in order for them to converge properly, and frequently multiple parameter sets could fit the data equally well. Therefore, all subsequent analyses used the power-law model.

(Part 3) Test for effect of initial aspirated length on apparent compliance

Table S1: ANCOVA table: Compliance J[t] versus initial aspirated length (L[0])

	Loading suction experiment		Stress application rate experiment	
Factor*	J[60]	J[300]	J[60]	J[300]
L[0]	P=1 ($F_{1,11}$=0.000)	P=0.8 ($F_{1,11}$=0.390)	P=0.3 ($F_{1,10}$=1.34)	P=0.07 ($F_{1,10}$=4.05)
Clutch	P=0.9 ($F_{5,11}$=0.235)	P=0.8 ($F_{5,11}$=0.397)	P=0.07 ($F_{4,10}$=3.06)	**P=0.01* ($F_{4,10}$=5.96)**
Clutch*L[0]	P=1 ($F_{5,11}$=0.128)	P=1 ($F_{5,11}$=0.144)	P=0.1 ($F_{4,10}$=2.66)	**P=0.02* ($F_{4,10}$=4.78)**

*Clutch was treated as a random factor, while loading pressure was treated as a linear covariate.

(Part 4) Reconstructing pressures from displacements

To calculate pressure changes from displacements we first used the initial response to the tissue to the load pressure to calculate parameters A and β of the power-law viscoelastic model for creep compliance (J(t); see main text equation 2). We then used these parameters to extrapolate what the tissue position would have been at later time points if there had been no further pressure changes. We then calculated the deviations (m(t)) between the actual tissue position and the extrapolated tissue positions at each frame (t>300 s). These deviations can be represented by the vector **m**.

(eqn. S4.1) $$\mathbf{m} = \begin{bmatrix} m(t_1) \\ m(t_2) \\ \vdots \\ m(t_n) \end{bmatrix}$$

We approximated pressure changes at each time point (ΔP(t)) as a series of small step functions associated with each frame in the time lapse sequence, giving a pressure change vector **ΔP**.

(eqn. S4.2) $$\Delta\mathbf{P} = \begin{bmatrix} \Delta P(t_0) \\ \Delta P(t_1) \\ \vdots \\ \Delta P(t_{n-1}) \end{bmatrix}$$

The creep compliance (J(t)) can be put into a discrete form as the matrix **J**:

(eqn. S4.3) $$\mathbf{J} = \begin{bmatrix} J(t_1 - t_0) & 0 & \cdots & 0 \\ J(t_2 - t_0) & J(t_2 - t_1) & & \\ \vdots & & \ddots & \\ J(t_n - t_0) & & & J(t_n - t_{n-1}) \end{bmatrix}$$

Discretizing the pressure, the deviations in position, and compliance in this manner gives the following equation for the deviations in position due to the unknown pressure changes:

(eqn. S4.4) $\mathbf{m} = -kR_c\mathbf{J}\Delta\mathbf{P}$

By taking the inverse of **J**, we can solve for the pressure changes at each time point necessary to drive the observed deviations in tissue position:

(eqn. S4.5) $$\frac{-\mathbf{J}^{-1}\mathbf{m}}{kR_c} = \Delta\mathbf{P}$$

The pressures at any given time-point are the sum of the previous pressure changes, so equation S4.5 allows extraction of applied pressure pulses, or unknown equivalent pressures (supplemental text part 6) from observed tissue positions.

(Part 5) Electrically induced contractility

To characterize the dependence of contractions on stimulus current (Fig. S1A) we applied stimuli ranging from 0.27 to 8.7 µA (Fig. S1B), with 4 stimuli of different intensities given to each embryo at 7 min intervals. Contraction magnitude appeared to increase smoothly with stimulus strengths above 0.4 µA, without an obvious threshold. The amount of contraction appeared to level off starting around 1.6 µA. Blebs were often observed forming on the surface of aspirated tissue after applying stimuli ≥1.6 µA, but were less often observed for weaker stimuli.

Since we wished to use the lowest intensity of stimulus that could consistently induce strong contractions we tested stimuli from a narrower range currents (1.6, 2.6, and 4.6 µA) near the shoulder of the curve. Three stimuli were given to each embryo with 7 minutes between stimuli. The magnitude of the first stimulus given to each embryo in a clutch was chosen at random, but without replacement, so that each embryo within a clutch received a different current for the first stimulus.

Focusing on the first stimulus given to each embryo in the second experiment, we found that the contractions increased with stimulus intensity (Table S2), but that a two-fold change in intensity (centered on 2.6 µA) would only produce a 50 % change in contraction strength (Fig. S1C). Two additional stimuli were given to each embryo at 7 min intervals after the first stimulus at different intensities. Contractions produced by subsequent stimulations of the embryo produced contractions within the same range of magnitudes as the contractions produced by the first stimulus. Based on these results, our subsequent experiments with electrical stimulation used 2.6 µA stimulus since this appeared to be the lowest current that produced a strong contraction.

We suspected variability in the electrical properties of the embryo or of the seal around the channel opening might contribute to variability in the current passing through the epithelium. To assess variability in the electrical properties of the embryo and seal, we measured the difference in resistance between the open channel and the channel with an embryo pressed to the opening to estimate the added resistance of the embryo. We measured the resistance of the channel (with or without the embryo) using Ohm's law since pulses were approximately square. We used an oscilloscope to measure voltages across the 505 kΩ resistor in series with the channel and the 10kΩ resistor in parallel with the channel.

We compared the resistance between unwounded embryos at high suction and at low suction, and between wounded and unwounded embryos at high suction (Fig. S1D). The coefficient of variation in resistance was only 10% (n=6 embryos for each condition), and there was negligible difference in resistance between unwounded embryos at high suction (0.40 ± 0.03 MΩ, mean ± SD), unwounded embryos at low suction (0.39 ± 0.04 MΩ), and wounded embryos at high suction (0.37 ± 0.04 MΩ) and unwounded embryos. Hence, variation in the electrical seal and embryo resistance was negligible.

Table S2: ANCOVA for displacement versus current* for the first pulse.

**Current**	**Clutch**	**Current*Clutch**
**P = 0.015 ($F_{1,4}$ = 16.6)**	P = 0.6 ($F_{2,4}$ = 0.6)	P = 0.13 ($F_{3,4}$ = 3.5)

***Current was treated as a linear covariate; clutch was treated as a random factor.**

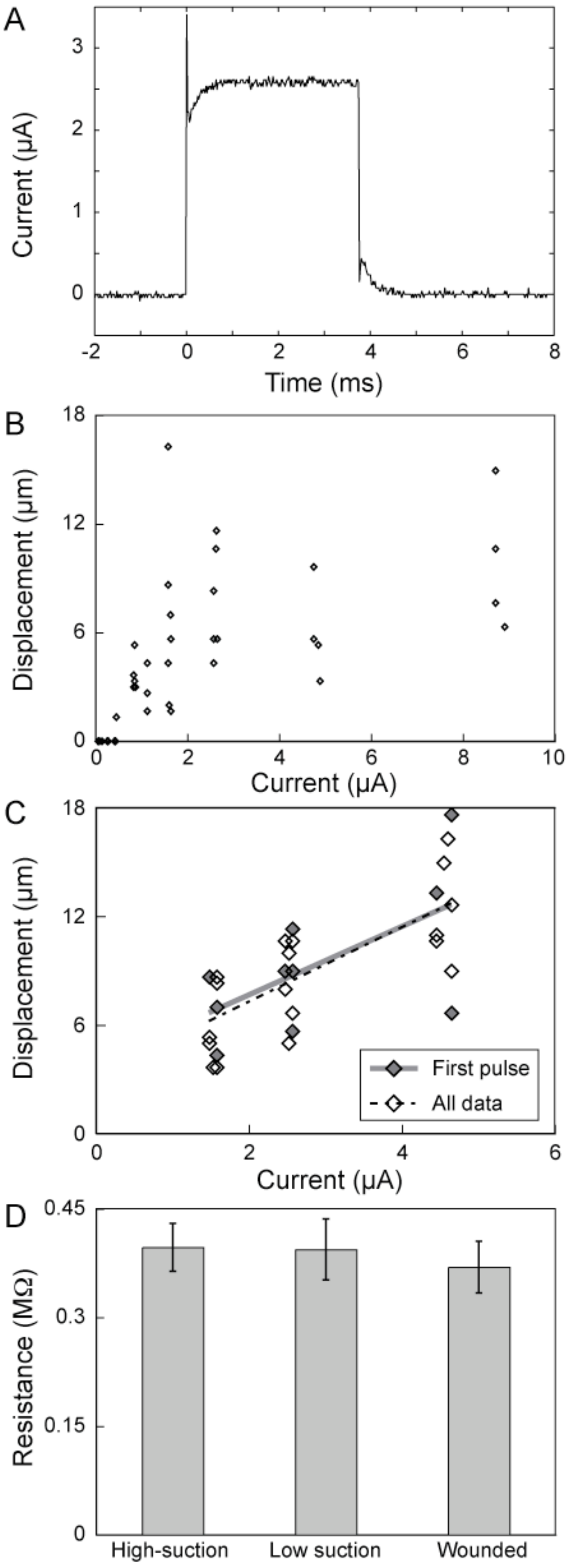

Supplemental figure 1: Refining electrical simulation of contractions. A) A current pulse. B-C) Tissue displacement versus stimulus current. Embryos in B were stimulated four times over a broad range of currents. Embryos in C were stimulated once at each of three currents. Filled symbols indicate the data from the first stimulus applied to each embryo. Linear least squares fits shown for clarity. D) Estimated electrical resistance of the embryo for embryos under low suction or high suction, or for wounded embryos.

(Part 6) Two models of tissue contractions

Here we develop two simple models of the mechanical forces driving induced contractions.

S6.1. Apical contraction: One way to get a contraction would be to have the apical surface of the epithelial layer contract against the resistance of the bulk tissue. Contraction of the apical surface can be treated as the development of surface tension over the aspirated tissue. The tissue displacements are driven by the sum of the applied suction (P_{ap}) and a pressure term generated by the surface tension (the equivalent pressure, P_{eq})

(eqn. S6.1.1) $$L(t) = -kR_c \int_0^t J(t-\gamma)\frac{dP_{ap}}{d\gamma}\,d\gamma - kR_c \int_0^t J(t-\gamma)\frac{dP_{eq}}{d\gamma}\,d\gamma$$

We can discretize equation S6.1.1 and use the same approach used to reconstruct pressure pulses (supplemental text part 4) to calculate the equivalent pressures as a function of time.

Assuming that the aspirated tissue approximates a spherical cap with radius of curvature r, the surface tension (T) would be related to the equivalent pressure by the law of Laplace:

(eqn. S6.1.2) $$P_{eq} = 2 \cdot T/r$$

Laplace's law is based on a force balance between the normal forces and the tension in the curved surface of a material. This force balance does not make any specific requirements on the material properties but it requires that shear stress components at the tissue surface are small enough to be neglected. Given our assumption that the surface tension is not present prior to the start of the contraction, and the fact that the tissue is

loaded by fluid pressure, there would be no shear stress at the tissue surface prior to contraction. Because the surface tension developed during the contraction would impose normal stresses on the tissue, we assume that shear stresses on the tissue surface would remain small during the contraction.

For a spherical cap, the radius of curvature would be given by the following:

(eqn. S6.1.3) $$r = \begin{cases} \dfrac{R_c^2 + L^2}{2 \cdot L} & 0 \le L \le R_c \\ R_c & L \ge R_c \end{cases}$$

To test whether this approximation was reasonable, we measured the surface curvature in those embryos with an aspirated length greater than the channel radius (using embryos from the experiment an varying load pressure). We measured the lensing effect of the PDMS channel by inserting a thin, pulled glass fiber into the channel, moving it across the channel, and observing the differences in the apparent displacement of parts of the end of the fiber inside the channel in comparison to the part of the fiber that was outside the channel. Lensing resulted in a uniform reduction in the apparent displacement of the fiber relative to the true displacement. To compensate, the images of the tissue were stretched by a factor of 1.06 in the direction perpendicular to the channel, but not stretched parallel to the channel. The curvature of the tissue surface at t = 300 s (just before the induced contraction) was measured by fitting a circle to the tissue surface by eye, attempting to minimize the deviations from the circle for all parts of the tissue that did not appear to be in contact with the channel wall. Based on equation S6.1.3, we expect $r = R_c$. The median radius of curvature was $1.14R_c$ (1st quartile: $1.09R_c$; 3rd quartile: $1.23R_c$), reasonably close to the predicted curvature.

Combining equations S6.1.2 and S6.1.3 gives the surface tension as follows:

(eqn. S6.1.4) $$T = \begin{cases} \frac{P_{eq}}{2} \cdot \frac{R_c^2 + L_c^2}{2 \cdot L_c} & 0 \le L \le R_c \\ \frac{P_{eq}}{2} \cdot R_c & L \ge R_c \end{cases}$$

For a fixed stimulus strength (fixed current pulse), we expect the changes in surface tension over time to be the same for any degree of aspiration, so we expect that the calculated surface tension to be constant for different levels of suction. Because the radius of curvature will decrease with suction pressure, this model also predicts that the equivalent pressure will increase with increasing suction. One caveat is that we cannot distinguish between purely apical contraction and contraction that is spread over some depth which is small in comparison to the channel radius.

S6.2. Isotropic contraction: If the tissue is compressible, another possible mechanism for contraction would be the development of uniform isotropic contractile stress throughout the tissue. Clearly, uniform isotropic contraction can only produce displacement if the material is compressible ($\nu<0.5$). To start with, we ignore viscoelasticity. Assuming that the embryo behaves as a linear, isotropic elastic material, and that deformations are small, we can take the relationship between Cauchy stress (σ) and Cauchy strain (ε) as:

(eqn. S6.2.1) $$\varepsilon_{ij} = \frac{1+\nu}{E}\sigma_{ij} - \delta_{ij}\frac{\nu}{E}\sum_{k=1}^{3}\sigma_{kk}$$

Here ν is the Poisson's ratio, E is the Young's modulus, and δ_{ij} is the Kronecker delta ($\delta_{ij}=0$ for $i \neq j$; $\delta_{ij}=1$ for $i=j$).

In this model, isotropic contraction modifies the stress tensor as follows:

(eqn. S6.2.2) $\sigma^*_{ij} = \sigma_{ij} + S\delta_{ij}$

Here σ^*_{ij} is the stress within the contracted configuration, and 'S' is the additional cell-generated stress during a contraction. We assume that the contracted tissue is also a linear, isotropic material, with a Poisson's ratio identical to the uncontracted material but, possibly, with a differing Young's modulus. Thus,

(eqn. S6.2.3) $\varepsilon^*_{ij} = \frac{1+\nu}{E^*}\sigma^*_{ij} - \delta_{ij}\frac{\nu}{E^*}\sum_{k=1}^{3}\sigma^*_{kk}$

Here ε^*_{ij} is the strain within the contracted configuration, and E* is the Young's modulus of the contracted material. Substituting (S6.2.2) into (S6.2.3), and then simplifying using (S6.2.1) we have:

(eqn. S6.2.4) $\varepsilon^*_{ij} = \frac{E}{E^*}\varepsilon_{ij} + \frac{1-2\nu}{E^*}S\delta_{ij}$

By definition, the infinitesimal strain is given by

(eqn. S6.2.5) $\varepsilon_{ij} = \frac{1}{2}\left(\frac{\partial u_i}{\partial x_j} + \frac{\partial u_j}{\partial x_i}\right)$ and $\varepsilon^*_{ij} = \frac{1}{2}\left(\frac{\partial u^*_i}{\partial x_j} + \frac{\partial u^*_j}{\partial x_i}\right)$

Here u_i and u_i* are the displacements in the x_i direction for the original and contracted configurations, respectively. The following expression satisfies equations S6.2.4 and S6.2.5:

(eqn. S6.2.6) $u^*_i = \frac{E}{E^*}u_i + \frac{1-2\nu}{E^*}Sx_i$

This can be shown by taking the derivative of u_i with respect to x_j in equation S6.2.6, and adding it to the derivative of u_j with respect to x_i.

A true strain tensor uniquely determines the displacement field, absent six degrees of freedom corresponding to rigid body motion. To satisfy the conditions that the tissue

does not rotate, move laterally across the channel opening, move into the solid material around the channel, or become detached from the channel opening we impose the following conditions on the displacements. If the origin is at the center of the channel opening, x_1 is taken as the direction along the channel axis, and x_2, and x_3 are perpendicular to the channel axis, then symmetry requires the following at the channel midline:

(eqn. S6.2.7) $$u_2 = u_2^* = 0 \qquad u_3 = u_3^* = 0$$

Furthermore, the conditions that the tissue neither detaches from the channel nor overlaps the material around the channel requires that at the edge of the channel opening:

(eqn. S6.2.8) $$u_1 = u_1^* = 0$$

If the applied suction were zero, the tissue could become detached from the opening. However, in the absence of body forces and inertia, any suction will hold the tissue onto the opening. These equations apply strictly only to infinitesimal deformations, so we examine small deformation caused by tissue contraction away from the suction-deformed configuration. As such, we take x_i from the suction-deformed configuration rather than the unstrained tissue.

In this coordinate system, applying the equation S6.2.6 to the displacement of the tip of the aspirated surface yields:

(eqn. S6.2.9) $$L^* = \left(\frac{E}{E^*} + \frac{1-2\nu}{E^*}S\right)L$$

L and L* are the aspirated lengths of the tissue before and during the contraction, respectively. Contraction of the embryo (S<0) causes the embryo to withdraw from the channel. However if the material is perfectly incompressible, $\nu = 0.5$ and the second term

vanishes, such that isotropic stress has no effect on the displacement. Increases in the Young's modulus also cause the embryo to withdraw from the channel, to a degree proportional to the ratio of the original and contracted Young's modulus.

Rearranging equation S6.2.9, and defining the deviation in position, m, due to contraction as L*-L gives:

(eqn. S6.2.10) $$\frac{m}{L} = \frac{L^* - L}{L} = \left(\frac{E}{E^*} + \frac{1-2\nu}{E^*}S\right) - 1$$

Since the load pressure does not appear in any of the terms on the right, the ratio of m to L on the left must be independent of pressure, even though L depends on pressure.

We could incorporate viscoelasticity and time dependent changes in contractility into equation S6.2.10 using the elastic-viscoelastic correspondence principle (Findley et al., 1989). However, because the load pressure term does not appear on the right, m/L must remain independent of load pressure according to this model.

Note also that because m goes to zero as L goes to zero, we also expect that the equivalent pressure will decrease as L decreases. This implies that the equivalent pressure will decrease with decreasing load pressure.

(Part 7) Finite element mesh

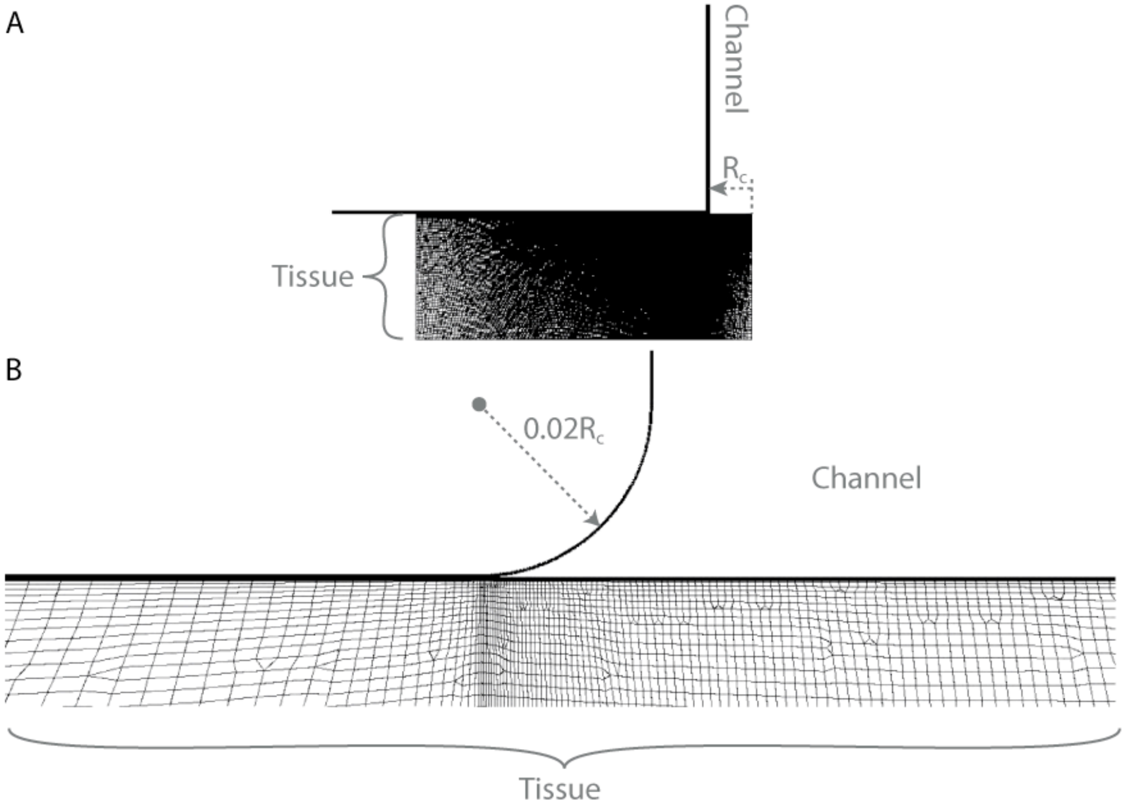

Supplemental figure 2: The un-deformed finite element mesh (A) and the portion of the un-deformed finite element mesh near the edge of the channel opening (B). R_c: channel radius.

Literature cited:

Boudou T, Ohayon J, Arntz Y, Finet G, Picart C, Tracqui P. 2006. An extended modeling of the micropipette aspiration experiment for the characterization of the Young's modulus and Poisson's ratio of adherent thin biological samples: Numerical and experimental studies. Journal of Biomechanics 39:1677-1685.

Findley WN, Lai JS, Onaran K. 1989. Creep and relaxation of nonlinear viscoelastic materials. New York: Dover Publications, Inc.

Merryman WD, Bieniek PD, Guilak F, Sacks MS. 2009. Viscoelastic properties of the aortic valve interstitial cell. J Biomech Eng 131:041005.

Sato M, Theret DP, Wheeler LT, Ohshima N, Nerem RM. 1990. Application of the micropipette technique to the measurement of cultured porcine aortic endothelial cell viscoelastic properties. J Biomech Eng 112:263-268.

Theret DP, Levesque MJ, Sato M, Nerem RM, Wheeler LT. 1988. The application of a homogeneous half-space model in the analysis of endothelial cell micropipette measurements. J Biomech Eng 110:190-199.

von Dassow M, Davidson LA. 2009. Natural variation in embryo mechanics: gastrulation in Xenopus laevis is highly robust to variation in tissue stiffness. Dev Dyn 238:2-18.

Zhou EH, Lim CT, Quek ST. 2005. Finite Element Simulation of the Micropipette Aspiration of a Living Cell Undergoing Large Viscoelastic Deformation. Mechanics of Advanced Materials and Structures 12:501 - 512.
